# Responsive Microneedles as a New Platform for Precision Immunotherapy

**DOI:** 10.3390/pharmaceutics15051407

**Published:** 2023-05-04

**Authors:** Xinyang Liu, Haohao Song, Tairan Sun, Hai Wang

**Affiliations:** 1 Henan Institutes of Advanced Technology, Zhengzhou University, Zhengzhou 450052, China; 2 CAS Key Laboratory for Biomedical Effects of Nanomaterials & Nanosafety, CAS Center for Excellence in Nanoscience, National Center for Nanoscience and Technology, Beijing 100190, China; 3 The Second Affiliated Hospital of Hebei North University, Zhangjiakou 075100, China; 4 University of Chinese Academy of Sciences, Beijing 100049, China

**Keywords:** microneedles, responsiveness, immunotherapy, controlled release, vaccine delivery

## Abstract

Microneedles are a well-known transdermal or transdermal drug delivery system. Different from intramuscular injection, intravenous injection, etc., the microneedle delivery system provides unique characteristics for immunotherapy administration. Microneedles can deliver immunotherapeutic agents to the epidermis and dermis, where immune cells are abundant, unlike conventional vaccine systems. Furthermore, microneedle devices can be designed to respond to certain endogenous or exogenous stimuli including pH, reactive oxygen species (ROS), enzyme, light, temperature, or mechanical force, thereby allowing controlled release of active compounds in the epidermis and dermis. In this way, multifunctional or stimuli-responsive microneedles for immunotherapy could enhance the efficacy of immune responses to prevent or mitigate disease progression and lessen systemic adverse effects on healthy tissues and organs. Since microneedles are a promising drug delivery system for accurate delivery and controlled drug release, this review focuses on the progress of using reactive microneedles for immunotherapy, especially for tumors. Limitations of current microneedle system are summarized, and the controllable administration and targeting of reactive microneedle systems are examined.

## 1. Introduction

As a drug delivery tool that has developed rapidly in recent years, microneedles have been widely used in the delivery of various nucleic acids [1,2], small molecules [3,4,5,6], proteins [7,8,9], nanoparticles [10,11,12], and other payloads, proving that microneedles as a new type of drug delivery system have strong flexibility. In particular, a variety of immunotherapeutic agents delivered by microneedles have achieved a strong increase in the immune level in animal experiments, such as fully inactivated influenza virus [13], hepatitis B virus plasmid DNA [14], and *Bacillus anthracis* protein antigens [15]. Vaccines have long been considered one of the most cost-effective medical interventions to preventing and controlling disease [16]. With the COVID-19 pandemic, due to the rich technical potential of microneedles for vaccine delivery, how to achieve efficient and accurate immunotherapeutic agent delivery has come under intense scrutiny.

As one of the most important immune organs, the skin is an attractive target for immunomodulation [17]. Transcutaneous immunization (TCI) has been reported to induce a stronger immune response than has intramuscular injection, which has a dose disadvantage [18,19,20]. Therefore, the application of microneedles in TCI has received extensive attention.

In terms of immunotherapeutic agent delivery, compared with traditional intramuscular and subcutaneous injections, microneedles can deliver loaded immunotherapeutic agents to the epidermis and dermis, which include many antigen-presenting cells, including dermal dendritic cells and epidermal Langerhans cells. These cells have the ability to capture antigens and stimulate lymphocytes to induce an immune response [21]. Based on the above-mentioned advantages of transdermal drug delivery, research on microneedles is also very popular in the field of influenza prevention and treatment. Currently, there are many microneedle-based immunotherapies in preclinical or clinical development, such as influenza vaccines [22], a measles-rubella vaccine [23], a rabies vaccine [24], and a spinal cord gray matter vaccine [25]. Compared with traditional subcutaneous injections, microneedle-based immunotherapy has the following advantages: (1) minimal invasiveness and painless administration [26]; (2) capacity to carry immunotherapeutic agents in dry form, improving thermal stability and reducing storage or transportation costs; and (3) a high potential for self-administration without the need for any special equipment or applicators [27]. The skin is full of immune cells targeted by the immunotherapy, which produces stronger immune effects than does subcutaneous injection and reduces the immunotherapeutic agent dose. Clinical studies have shown that microneedle-based immunotherapy can induce immune effects comparable or even better than those of traditional subcutaneous injections [28,29,30]. Early microneedle-based immunotherapy has mainly consisted of hollow microneedles. For example, the injection microneedle used in Fluzone Intradermal (IDflu, Intanza) [31], a flu vaccine launched in 2011 with Sanofi, is BD’s Soluvia hollow microneedle delivery system [32]. However, such vaccines are still in liquid form, which is not conducive to storage or transportation. In order to solve this problem, a growing number of researchers have begun to focus on the development of dissolvable microneedle-based immunotherapy. There are the related studies on soluble microneedles in the development of various vaccines such as novel coronavirus, Ebola virus [33], AIDS virus, influenza, polio, and rabies [34]. Targeted delivery of immunotherapeutic agents to immune cells enables dose reduction and enhanced immune response [35,36,37]. Additionally, microneedle transdermal drug delivery has the advantages of high compliance, moderate invasiveness, and convenient use [38,39,40]. The development of a responsive microneedle system can further control the spatiotemporal release of payloads more effectively.

Stimulus conditions for responsive microneedles can be classified into internal and external stimuli according to where the stimulus occurs. In this review, we classify responsive microneedles for immunotherapy into the above two categories, and introduce the materials, structures, and functions of stimuli-responsive microneedles, as well as their applications in medicine and biology. All stimulus-triggering methods and diseases are summarized in the tables.

## 2. Microneedles Triggered by Internal Stimuli

### 2.1. pH-Responsive Microneedles

pH-responsive microneedles are mostly made of polymers [41,42]. Changes in the pH of the environment can cause the polymer to degrade, swell, rupture, or collapse to release the loaded immunotherapeutic agents. These materials typically consist of hydrophilic and ionically functional polymer chains. Low pH has been intensively studied as a significant factor in the development of several pH-responsive microneedle-based drug delivery systems at the sites of chronic wounds, inflammatory processes, and cancer [43,44,45,46]. For example, the pH of most tumor microenvironments is lower than that of normal tissues, so this responsive approach has important practical value in cancer immunotherapy [47,48,49,50,51,52].

**Figure 1:**
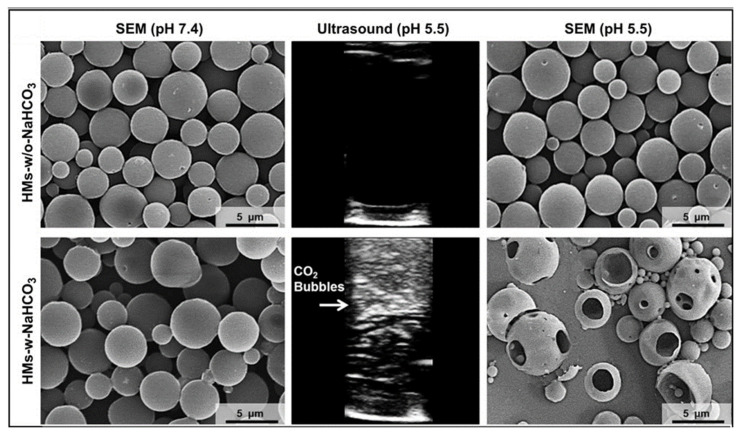
Both unloaded and loaded NaHCO_3_ microspheres were intact at pH 7.4. In the acidic environment of pH 5.5, the microspheres containing NaHCO_3_ were swollen by CO_2_ gas and a large number of holes were ruptured. Reprinted with permission from [54]. Copyright 2012, Elsevier.

In a previous study, a pH-responsive drug delivery system was developed by encapsulating hollow microspheres of lactic acid–glycolic acid copolymers in polyvinylpyrrolidone (PVP) microneedles [53,54]. Two drugs were used for simultaneously delivery. The PVP microneedles loaded with the first drug and hollow microspheres dissolved immediately after being inserted into the skin for a few minutes, releasing the first drug and hollow microspheres in the environment. In the acidic pH skin environment, protons (H^+^) could freely and quickly pass through the PLGA shell membrane, react with NaHCO_3_ in the microspheres, and generate a large number of CO_2_ bubbles (Figure 1). These bubbles created holes and ruptured the PLGA spherical shell membrane, thereby releasing the second encapsulated drug in the microspheres. This approach could become a powerful technique for the continuous direct transdermal delivery of multiple drugs into the epidermis for clinical applications.

**Figure 2:**
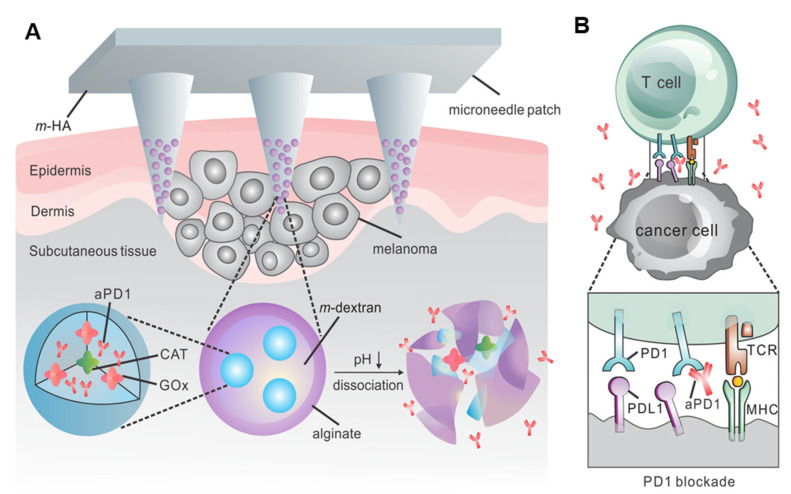
Schematic diagram of the microneedle-responsive patch delivery of aPD1 for skin cancer treatment. (**A**) Two enzymes are encapsulated in the nanoparticles. Under the action of enzymes in the physiological environment, blood glucose is converted into gluconic acid to promote the continuous dissociation of NPs, thereby releasing aPD1. (**B**) aPD1 blocks PD-1, and the activated immune system kills skin cancer cells. Reprinted with permission from [56]. Copyright 2016 American Chemical Society.

In another study, a self-degradable biocompatible hyaluronic acid microneedle was loaded with pH-sensitive glucose nanoparticles that encapsulate antiprogrammed death 1 (aPD1) antibody and glucose oxidase (GOx), which converts blood glucose to gluconic acid (Figure 2A) [55,56]. The generation of an acidic environment facilitated the self-dissociation of nanoparticles, which subsequently resulted in a massive release of aPD1. Skin is a perfect tissue for triggering an associated immune response [57,58,59]. aPD1 acts as an immune checkpoint inhibitor to prevent T-cell apoptosis by blocking the PD-1 receptor on the surface of T cells [60]. This enables T cells to effectively fight tumor cells. In this work, pH-responsive dextran nanoparticles were prepared by a double-emulsion (oil-in-water) solvent evaporation/extraction method. Compared with systems without triggered release or intratumoral injection, pH-responsive microneedles had a more pronounced therapeutic effect in eradicating a mouse model of melanoma [61,62,63,64,65]. Overall, the microneedle delivery system has great potential and biomedical promise in the treatment of skin cancer.

**Figure 3:**
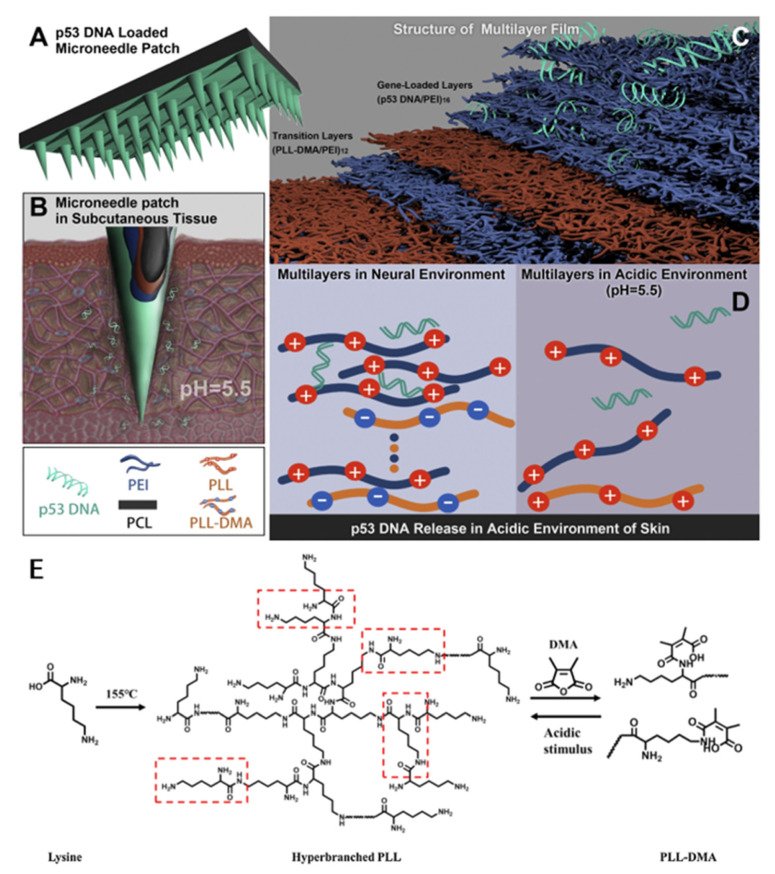
Scheme of a microneedle patch modified with pH-responsive transition layers and gene-loaded layers using layer-by-layer assembly. (**A**–**D**) The two layers of a layered structure are composed of a pH-sensitive transition layer and a gene-loaded layer. When a microneedle patch is placed on the skin, the microneedle coating contacts the skin microenvironment, the negatively charged portion of the PLL-DMA will transform into a positively charged one. The transition layer will then collapse, which will accelerate the release of the outermost p53 DNA. (**E**) The synthetic path of PLL and PLL-DMA. Reprinted with permission from [66]. Copyright 2019, Elsevier.

Unlike the above two works that used microneedles to encapsulate pH-responsive nanoparticles, the following work developed a microneedle responsive to cancer acidic skin [48,66]. A pH-responsive polyelectrolyte multilayer film (PEM) was coated on the surface of polycaprolactone (PCL) microneedles using a stepwise multilayer assembly for gene release in skin cancer. The PEM consists of a transition layer, a charge-reversible polymer, a dimethylmaleic anhydride-modified polylysine (PLL-DMA), and a gene-loading layer (p53 expression plasmid/polyethyleneimine; Figure 3). When the microneedle coating contacts the intradermal microenvironment, the negatively charged PLL-DMA becomes a positively charged PLL due to the low pH environment. Rapid dissolution of the transition layer accelerates the release of the p53 DNA in outermost layer. The results of in vivo experiments showed that the pH-responsive microneedles could effectively inhibit tumor growth in mice, and the tumor mass of nonresponsive microneedles or mice injected intravenously was 5 to 10 times that of pH-responsive microneedles.

**Figure 4:**
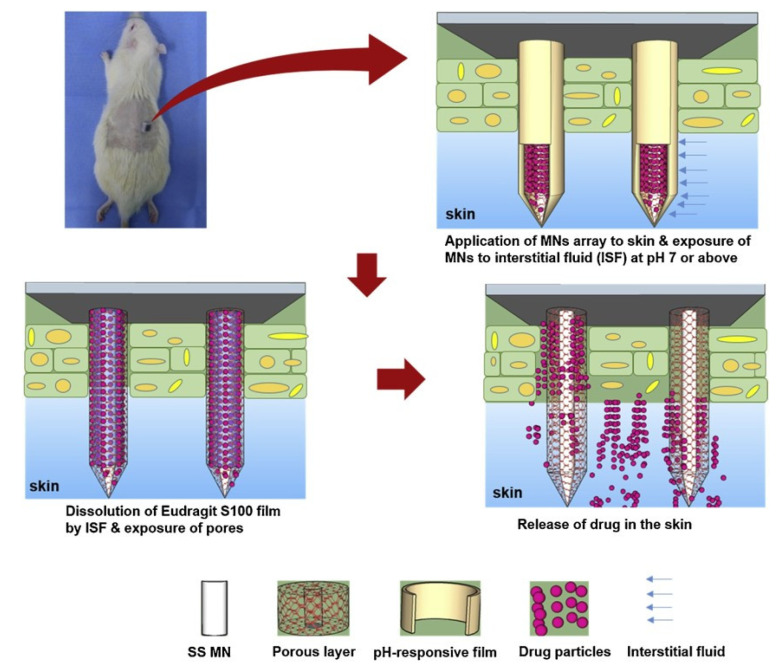
After the microneedles are exposed to the medium at alkaline pH (vulnus pH), the drug encapsulation film is dissolved, and the pores on the microneedle are exposed, which releases the encapsulated payload in the vulnus pH microenvironment. Reprinted with permission from [67]. Copyright 2021, Elsevier.

Lastly, a pH-responsive wound coating was proposed on porous polymers, which covered the pores of drug-filled porous microneedles to prevent drug leakage and drug release in the wound pH environment (Figure 4). At the pH of healthy skin (4.5), drug release from the microneedles was negligible [67]. In contrast, significant release occurred in the wound pH environment. This kind of microneedle model that can be produced in large quantities was developed, and coating materials with different reaction conditions were combined with antibacterial agents and anti-inflammatory drugs to increase the treatment of wound infections.

### 2.2. Reactive Oxygen Species-Responsive Microneedles

Reactive oxygen species (ROS) are important signals for biological metabolism and intercellular communication. In various pathological processes such as diabetes, inflammation, aging, neurodegenerative diseases, and cancer, ROS levels are significantly higher than normal due to metabolic abnormalities [68,69,70,71,72,73]. By exploiting the difference in ROS levels between normal and diseased tissues, various ROS responsive groups have been developed for ROS-specific responsive drug delivery systems. Currently, ROS-responsive microneedles are mainly used for the treatment of diabetes. We focus on the application of ROS-responsive microneedle-based immunotherapy design for the prospect of being applicable to other diseases.

Commonly used ROS-responsive polymers have two main responsive mechanisms. One is the change of hydrophilicity and hydrophobicity under the action of ROS, and the other is the cutting change of structure affected by ROS [74,75,76,77,78]. For one, ROS can oxidize chalcogenide elements and increase their valence. During this process, oxygen and sulfur atoms form covalent bonds, and polar groups form hydrogen bonds with ambient water molecules, thereby initiating a hydrophobic–hydrophobic–hydrophilic transition of the polymer backbone without disrupting the polymer’s chemical structure [79]. For another, ROS can react with chemical structures such as thioketals (TKs), phenylboronic acids/esters (PBAs/PBEs), vinyl disulfides, and proline oligomers, resulting in the cleavage of these structures [80]. A variety of ROS-responsive microneedles were designed through these two mechanisms.

**Figure 5:**
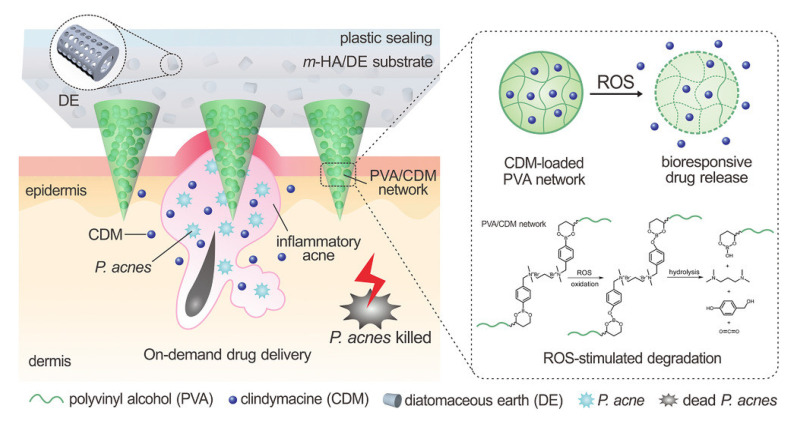
The mechanism diagram of a microneedle patch responding to ROS in an acne pathological environment. In order to adsorb pus and cell debris and their necrotic cell by-products, diatomite (DE) was used as one of the materials. For more softness and better biocompatibility, methacrylic acid esterified hyaluronic acid was used. Both promote wound healing and prevent potential recurrence. Drug-responsive polyvinyl alcohol (RR-PVA)-ROS was released on demand under pathological conditions to reduce side effects. Reprinted with permission from [81]. Copyright 2018, John Wiley and Sons.

Authors have proposed a new drug delivery method for antiacne treatment using ROS-responsive microneedle patches (Figure 5) [81]. This method avoids the risk of destroying intestinal flora and other risks caused by oral antibiotics or isotretinoin. Compared with commonly used acne creams, microneedles can enhance the efficacy of dermal lesions through skin penetration. Controlled and sustained release of drugs to counter the overproduction of reactive oxygen species in acne is also important for improving antimicrobial efficacy and reducing side effects. Therefore, microneedles were fabricated from drug-loaded ROS-reactive poly (vinyl alcohol) (RR-PVA) matrices to achieve on-demand drug release and reduce side effects. In addition, a matrix containing methacryloyl hyaluronic acid (m-HA)/diatomaceous earth (DE) was used as a support matrix for the microneedles, which can absorb pus and other purulent exudates and debris to promote healing, potentially preventing future relapse. The related study shows that this ROS-responsive microneedle patch can effectively transfer antibiotics to the dermis and can also attach functions such as adsorbing harmful substances in the wound to eliminate acne in a convenient manner.

### 2.3. Enzyme-Responsive Microneedles

**Figure 6:**
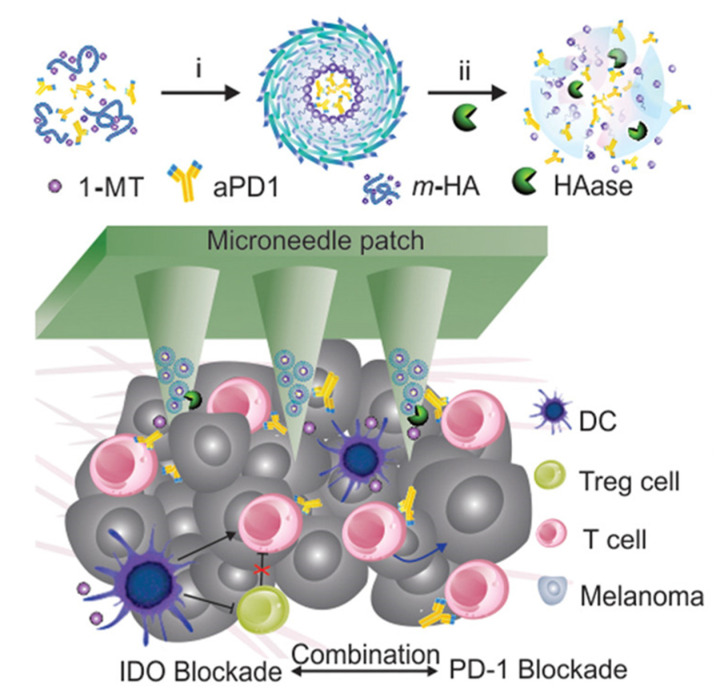
A schematic diagram of the hyaluronic acid enzyme-response microneedle patch combined with immune adjuvant therapy. The self-assembled nanoparticles (NP) of IDO and aPD1 were dissociated from the microneedles under the action of HAase and combined with DC cells and T cells to exert immunotherapy. Reprinted with permission from [88]. Copyright 2016, American Chemical Society.

Enzymes are ubiquitous in metabolic reactions in the body and play a vital role in healthy metabolic homeostasis. When diseases occur, the function of enzymes and the content of specific enzymes are mostly abnormal, especially in tumors. It was found that the expression level of enzymes is correlated with the stage of cancer development at all stages of cancer. Because of the high availability of hyaluronidase, it has been used as a key target in many works to design enzyme-responsive drugs [82,83,84,85]. The overexpression of hyaluronidase in the tumor microenvironment is used to achieve enzyme-responsive drug release in the tumor area [86,87]. In one work, hyaluronic acid-loaded anti-PD1 antibody (aPD1)-entrapped immunotherapeutic nanoparticles were combined with the IDO inhibitor 1-methyl-DL-tryptophan (1-MT) to assemble nanoneedles (Figure 6) [88]. The designed enzyme-responsive microneedle drug delivery device could trigger sustained release and enhance the retention of checkpoint inhibitors in the tumor microenvironment. Compared with the absence of hyaluronidase, aPD1 and 1-MT release increased to 4 and 2 times in the presence of hyaluronidase, respectively. This enzyme-responsive microneedle produced enhanced T-cell immunity in a B16 mouse melanoma model, alleviated local immunosuppression, effectively inhibited melanoma growth in mice, and contributed effectively to antitumor immunotherapy.

In addition to tumor immunotherapy, enzyme-responsive microneedles also have outstanding applications in antibacterial therapy. Studies reported that the expression of gelatinase at the wound site was significantly upregulated [89,90], indicating favorable conditions for the use of gelatinase-responsive microneedle for antimicrobial therapy and wound healing. X Lei et al., designed a degradable microneedles patch made of physically inert polymer PVP K-30 and recombinant type III collagen, which released antibacterial peptides encapsulated in gelatin nanoparticles in the presence of overexpressed gelatinase at the wound, achieving antibacterial treatment and healing of chronic wounds as well as remarkable therapeutic effects in mouse models [91].

The advantage of enzyme-responsive microneedle systems is that they can utilize the differences in enzyme expression levels between the pathological microenvironment and the normal physiological environment to achieve specific treatment and minimize the potential toxicity of drugs to normal cells and tissues. Nevertheless, it is difficult to achieve a complete controlled release of drugs based on enzyme response alone, and there is the possibility of the early release of drugs. Therefore, the combination of enzyme response with other response methods will effectively improve the utilization efficiency of drugs.

### 2.4. Temperature-Responsive Microneedles

**Figure 7:**
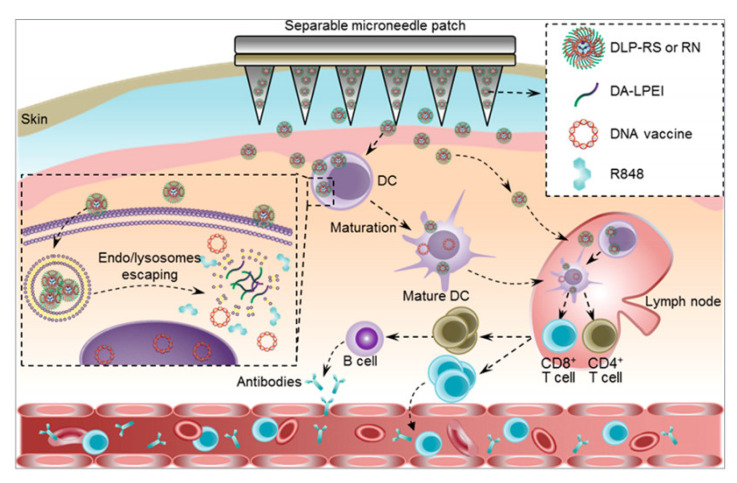
Schematic illustration of the separable microneedle patch mediated intracutaneous delivery of DNA nanovaccines for fighting the SARS-CoV-2 virus. Deoxycholic acid-conjugated low-molecular weight polyethylenimines (DA-LPEI) was applied to encapsulate both R848 and S-or N-protein-encoding DNA vaccines (DLP-RS or RN). The microneedle patch can painlessly penetrate the epidermis into the dermis layer, where it releases the DLP-RS or DLP-RN nanoparticles. Reprinted with permission from [99]. Copyright 2021, American Chemical Society.

Temperature response is an important research aspect of smart biomaterial response. Temperature-responsive microneedles utilize the property of phase change of heat-sensitive polymers according to the body temperature to achieve the controlled release of drugs. At present, a variety of heat-sensitive polymers have been used for the preparation of temperature-responsive microneedles [92,93,94]. The body surface temperature is around 33–37 °C. Under some pathological conditions, the skin temperature will increase or decrease depending on the type and process of disease [95]. Therefore, the change of body surface temperature according to the pathological conditions can be used as an effective stimulus for the temperature-responsive microneedles to release drugs. The traditional microneedle systems need the patch to adhere to the skin for a long time to achieve sustained drug release, which poses a challenge to the adhesion of microneedle patch. Many studies have focused on enhancing the adhesion effect by changing the morphology of the microneedles or replacing the substrate material [96,97]. However, some studies have considered the controllable separation of the tip and the substrate by adding a heat-sensitive polymer in the microneedle system so as to achieve more reliable sustained drug release [98,99,100]. Yue Yin and colleagues used temperature response in developing a separable microneedle system capable of delivering DNA vaccines and immune adjuvants [99]. Firstly, deoxycholic acid conjugated low-molecular weight polyethylenimines (DA-LPEI) nanoparticles coated with an immune-stimulating agent R848 are prepared, a DNA vaccine is then adsorbed on the surface of the nanoparticles via electrostatic interaction, and finally the nanoparticles are encapsulated at the tip of a microneedle. PNIPAM-B is added between the tip and a PVA substrate as a separation layer. PNIPAM-B has a low critical solution temperature (14–16 °C), is insoluble in water at room temperature (~22 °C), and is highly soluble at or below 14 °C. After the microneedles are embedded into the skin, the tip and the substrate can be separated by reducing the skin temperature to 14–16 °C for several minutes, with the tip being retained in the dermis layer to continuously release the DNA vaccine. The results showed that the microneedles could stably store the DNA vaccine at room temperature for up to 30 days, and the tip could be effectively separated from the substrate by temperature control, resulting in a durable and strong antiviral immune response (Figure 7).

For certain temperature-sensitive and fragile active drugs such as mRNA, achieving effective in vivo delivery is environmentally demanding, and it is delivered mainly via intramuscular injection, which usually causes pain. Moreover, conventional microneedles cannot meet the temperature-sensitive requirements for the delivery of active drugs. Meanwhile, the materials used to prepare the microneedles must meet certain strength and hardness requirements to achieve a successful puncture, which limits the selection of materials and thus restricts the application of the microneedles. Therefore, cryomicroneedle technology has arisen at a historic moment.

Cryomicroneedles are capable of delivering a variety of active substances based on aqueous materials without impairing the activity of biological substances, with the tip remaining in the skin to continuously release the drug after the ice base melts under the influence of body temperature. Xiaoxuan Zhang et al., selected various types of materials, including water, temperature-curable materials, light-curable materials, and ion-crosslinked cured materials, which achieved significant strength enhancement after freezing and enabled effective delivery of small-molecule drugs (RhB), macromolecular drugs (fluorescein isothiocyanate-labeled BSA, erythropoietin), and microorganisms (bacillus subtilis) [88]. Jinming Yu et al., mixed mRNA with HA solution and prepared cryomicroneedles which can effectively puncture the skin by freezing [2]. Compared with PEI or liposome carrying the mRNA, the freezing operation did not affect the transfection efficiency of mRNA in HA microneedles. Specific B-cell and T-cell immune responses were successfully induced in mouse experiments. Although there are still some challenges to be solved, such as disinfection methods and dose control, the flexibility of carrying active drugs on cryomicroneedles is still encouraging for the development of a new generation of microneedles [101].

As shown in Table 1, in addition to the work specifically described above, we also outline several representative responsive microneedles triggered by internal stimuli, outlining their microneedle design methods. It is hoped this can inspire methods and ideas for the comprehensive design of microneedle-based immunotherapy.

## 3. Microneedles Triggered by External Stimuli

In this section, we introduce the functions and structures of responsive compounds for externally triggered responsive microneedles and their biomedical applications.

### 3.1. Optical-Activated Microneedles

Optical triggering has the advantages of noninvasiveness, high spatial resolution, and temporal controllability [105,106]. Light-activated nanomaterials for cancer phototherapy include photothermal conversion materials, photosensitizer materials, and nanoplatforms containing photo-responsive parts [107,108,109]. Photothermal conversion nanomaterials are a type of material that can absorb light energy and convert it into heat energy leading to the death of cancer cells [110]. In general, photothermal conversion nanomaterials can be divided into organic materials and inorganic materials. Organic photothermal conversion nanomaterials can be synthesized by modifying organic dye molecules (i.e., indocyanine green; ICG) or using semiconductor polymers to prepare nanoparticles. Inorganic photothermal conversion nanomaterials include noble metal materials (i.e., gold nanomaterials), transition metal sulfides and oxides (i.e., copper sulfide nanoparticles), or carbon-based materials (i.e., graphene oxide). Using these materials to design controllable, light corresponding microneedles can realize the synergistic effect of chemotherapy and photothermal therapy (PTT) in tumor treatment.

**Figure 8:**
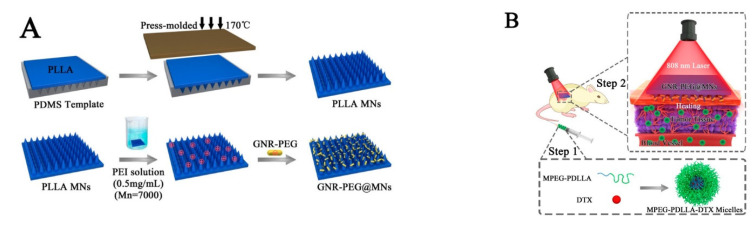
(**A**) The schematic diagram of the preparation process of PLLA microneedles and GNR-PEG@MN. (**B**) GNR-PEG@MN and MPEG-PDLLA-DTX micelles respond to near-infrared light and combined with chemotherapy to synergistically treat A431 tumors. In the first step, DTX micelles were injected. In the second step, microneedles were pressed on the tumor site and irradiated with an 808 nm laser at a power of 2 W/cm^2^ for 5 min. Reprinted with permission from [114]. Copyright 2017, American Chemical Society.

PTT is a promising therapeutic approach, with minimal invasiveness and good curative effect in cancer treatment. Under the irradiation of an external laser, these materials absorb light energy and convert it into heat energy, thereby killing tumor cells [111,112,113]. In one study, a near-infrared (NIR) responsive polyethylene glycol gold nanorod (GNR-PEG) coated poly (L-lactide) microneedle system (GNR-PEG@MN) was developed to improve the antitumor efficiency of docetaxel-loaded MPEG-PDLLA (MPEG-PDLLA-DTX) micelles for the treatment of A431 tumors (Figure 8) [114]. This system showed excellent heating ability both in vitro and in vivo. Moreover, GNR-PEG@MN has good skin insertion ability (with a height of 480 μm) and excellent biosafety. GNR-PEG@MN also exhibited good heat transfer ability in vivo, and the temperature of tumor site reached to 50 °C within 5 min. Compared with only chemotherapy or PTT treatment, this combination of GNR-PEG@MNs and MPEG-PDLLA-DTX micelles completely inhibited the growth of A431 tumors in vivo without recurrence, showing a significant synergistic effect.

### 3.2. Mechanical Force-Responsive Microneedles

**Figure 9:**
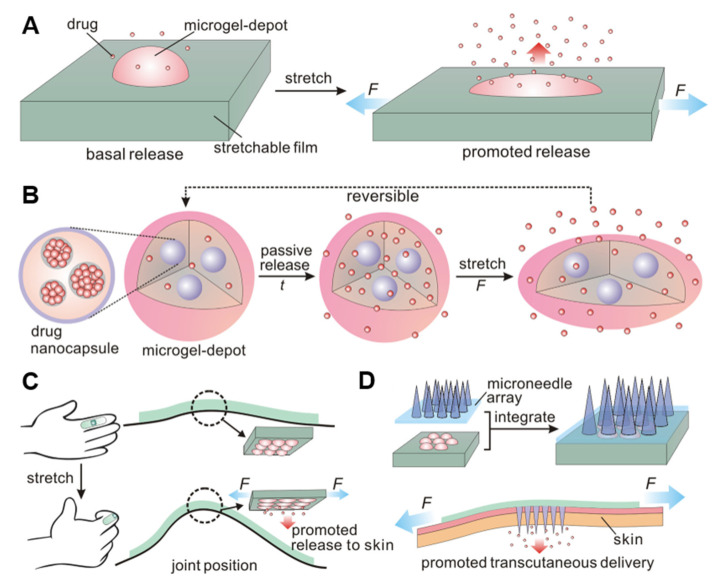
Diagram of the mechanical force response delivery system. (**A**,**B**) The elastomeric substrate deforms under stretching, releasing the drug from the microreservoir. (**C**) The drug-loaded patch is attached to the finger joint, and the drug release is triggered by bending the finger. (**D**) The drug-loaded patch is combined with the microneedle array patch, and the drug released from the drug-loaded library is transdermally administered through the microneedles. Reprinted with permission from [35]. Copyright 2015, American Chemical Society.

Stimulus-triggered drug delivery systems can achieve controlled release of dose, space and/or time and add many entry points for the design of a sustained release system. Many “smart” formulations developed thus far have been based on multiple stimuli, which can be physiological internal stimuli, such as ROS, redox potential, PH, ATP, and enzyme activity, or external physical triggers, such as NIR, thermal, ultrasound, and magnetism. Meanwhile, the method of triggering drug release based on mechanical forces such as stretching, pressing, and rotation is often transformed into clinical practice. Due to individual differences and complex physiological environments, it is impossible to accurately control the drug release dose, and the external trigger-mediated method is limited by additional instruments. Mechanical strain-based stimuli, accompanied by frequent daily motion, can provide a reliable and easy way to promote drug release in space and time (Figure 9) [35]. This strain change can be easily achieved through skeletal muscle elongation and shortening as well as bone, tendon, and joint cooperation. Furthermore, strain-triggered delivery systems are expected to release analgesics or rescue drugs from self-administration through simple body movements.

Microneedle systems based on mechanical force response are roughly divided into two types. One type functions to achieve controlled drug release by increasing the specific surface area of elastic microneedles through mechanical stretching. For example, the stretchable microneedle system developed by J Di and colleagues consists of hydrogel microneedles containing nanoparticles loaded with drugs on an elastic substrate [115]. The drugs are rapidly released in response to stretching by mechanical forces. However, this system has certain problems in accurate drug delivery because the quantitative relationship between the tensile force and the responsive release is difficult to control. The other type involves separating the microneedle tip from the substrate by mechanical stretching. Most microneedles systems face the problem that the microneedle patch easily falls off the skin before completing the release of drugs. The separation of the microneedle tip and the substrate through mechanical force response can solve this problem well. The rapid response microneedle patch developed by the Hyesun Jun team consist of a soluble HA tip and an insoluble PCL substrate [116]. After the microneedles are inserted into the skin, the HA tip is quickly separated from the substrate by the piercing mechanical force and remains in the skin to continuously release the drug.

Although there are few self-developed miniature-controlled drugs delivery systems mainly using pressure, these systems still provide us with a free, controllable, and convenient method of drug delivery. Combined with microneedles triggered by mechanical force, different trigger strengths and trigger angles can be designed to facilitate controllable and autonomous drug delivery. Table 2 summarizes the construction, payload, and application of representative responsive vaccines triggered by external stimuli.

## 4. Responsive Microneedle for Cancer Immunotherapy

Responsive microneedle-based immunotherapies, which are triggered in multiple forms and combined with multiple therapies, are an outstanding representative of on-demand drug delivery technology, meeting the treatment expectations of improving treatment efficacy and reducing systemic side effects [128,129]. This type of therapy can overcome the shortcomings of traditional drug carriers, can accurately control the drug release site, and can increase the therapeutic effect against diseases [130]. The internal stimuli mentioned in this paper, as well as the various external stimuli such as magnetism, temperature, and light, are constantly being explored by designers based on these factors. However, since single-responsive nanodrug carriers are sometimes not highly sensitive to a single factor, researchers are now focusing on the design of dual- and multiresponsive nanodrug carriers, such as pH/reduction, pH/temperature, reduction/enzyme, or pH/reduction/enzyme. More importantly, responsive microneedle-based immunotherapy is friendly to the human body [126] and easy to develop and biodegrade [131].

Tumor immunotherapy is a promising strategy to preventing cancer proliferation by activating the immune system to fight tumors. Some biological vaccine preparations, such as Sipuleucel-T (autologous active immune cell therapy) [132,133,134,135], have been used clinically. Inhibition of tumor progression by inducing immune responses has become a research hotspot. In immunotherapy, checkpoint inhibitors, such as anti-PD-1 [136], have been widely introduced into clinical practice. As another immunotherapy-based strategy, tumor vaccine can also open up a new field for tumor immunotherapy because it can induce antigen-specific immune response and activate immune memory [137,138,139]. However, systemic toxicity and low immunogenicity limit its wide clinical application. In addition, The application of tumor vaccine is also limited by the off-target effect of cytokine induction and immune stimulation adjuvant. Fortunately, biomaterials such as polymers [140,141,142], scaffolds [14], liposome [143,144,145], microneedle, and others [53,146,147,148] provide a possible answer due to their valuable properties, including excellent biosafety and biometabolism, on-demand control of size, high load of immune-related components, and multiple sites of immune ligand binding. Therefore, various biocompatible carriers are being used in tumor vaccines to enhance the immunogenicity of tumor antigens so that antigens can effectively and freely exert antigen presentation [149,150,151]. Combined with the above-mentioned responsive materials, microneedles can improve immune responses of various cancer immunotherapies. Here, we will briefly outline several representative works on responsive microneedles cancer immunotherapy.

**Figure 10:**
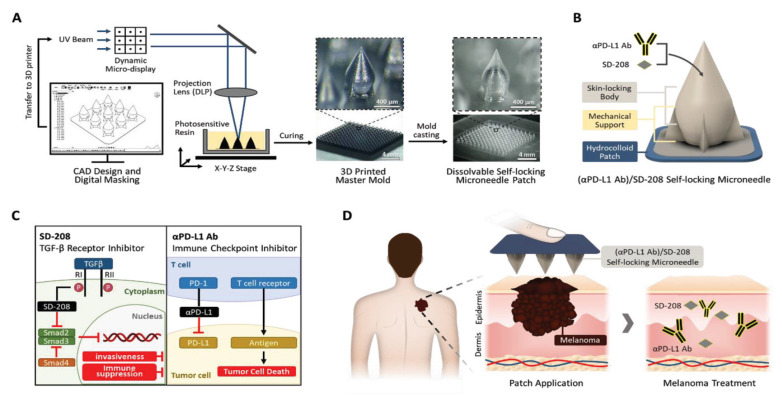
Fabrication of a self-locking microneedle patch. (**A**) The process of making the self-locking microneedle patch with a 3D printer based on surface projection microstereolithography technology. (**B**) The geometric structure of the self-locking microneedle: the sharp tip penetrates the skin, and the wide body and support structure at the bottom strengthen the bonding strength between the microneedle and the skin. (**C**) The mechanism of action of TGF-β receptor inhibitor SD-208 and immune checkpoint inhibitor αPD-L1Ab. (**D**) Schematic diagram of drug-loaded self-locking microneedles applied to melanoma. Reprinted with permission from [159]. Copyright 2023, John Wiley and Sons.

Traditionally, due to the limited machining capacity, dissolvable microneedles were mostly made into conical or polygonal conical geometric shapes, and the stability of microneedles entering the skin is insufficient [152,153,154,155,156,157]. One study overcame the limitations of the conventional microneedle. Self-locking microneedles fabricated by a novel projection microstereolithography 3D printer greatly increased the adhesive dose and transdermal drug delivery after insertion into the skin [158]. Self-locking microneedles attached to a flexible hydrogel patch, consisting of a narrow base with mechanical support, a sharp skin penetrating tip, and a wide skin interlock (Figure 10). This design greatly improves the accuracy of the microneedle’s penetration of irregular skin surfaces, especially for the irregular and uneven surface of melanoma skin. In terms of immune strategy, the combination of a new transforming growth factor-β (TGF-β) inhibitor (SD-208) and immune checkpoint anti-PD-L1 strongly inhibited tumor proliferation and metastasis, showing enhanced immunotherapy. Compared with that of intratumoral injection, the effective dose of self-locking microneedle-based immunotherapy to inhibit tumor is significantly lower.

**Figure 11:**
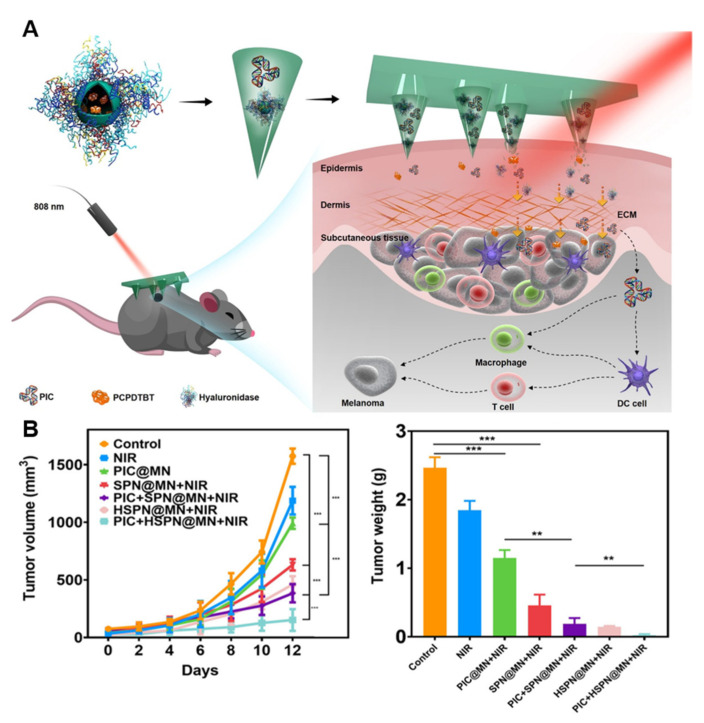
Photothermal microneedle for cancer immunotherapy. (**A**) Schematic of the microneedle system for synergetic cancer therapy. (**B**) Tumor inhibition effect of the mouse model. The statistical significance was assessed through one-way ANOVA statistical analysis or paired Student’s *t* tests: (**) for *p* < 0.01, and (***) for *p* < 0.001. Statistical significance was considered as *p* < 0.05. Reprinted with permission from [160]. Copyright 2021, American Chemical Society.

Due to the objective existence of the tumor matrix barrier, the killing effect of drugs and immune cells will be limited and cannot be deepened, thereby impairing the efficacy of cancer immunotherapy. One study reported an innovative synergistic PTT and immunotherapy [160]. Hyaluronidase and the semiconducting polymer poly(cyclopentadithio-phene-alt-benzothiadiazole) were used as microneedle substrates to deliver the immune adjuvant, polyinosinic acid (PIC). After the microneedles penetrated into the skin, hyaluronidase dissolved into the extracellular matrix, and semiconductor polymer nanoparticles and PIC were released into the tumor microenvironment (Figure 11A). Under laser irradiation, the microneedles interacted with PIC to activate immune cells, enhance T-cell immune response, and inhibit tumor growth and spread. For the in vivo experiments, hyaluronic acid-responsive microneedles encapsulated with semiconductor polymers and immune adjuvants showed excellent tumor growth inhibition ability (Figure 11B). This work developed an efficient microneedle platform that combines PTT with immunotherapy. This solved the obstacle of the tumor matrix barrier and effectively realized the immunotherapy of melanoma in a mouse model.

**Figure 12:**
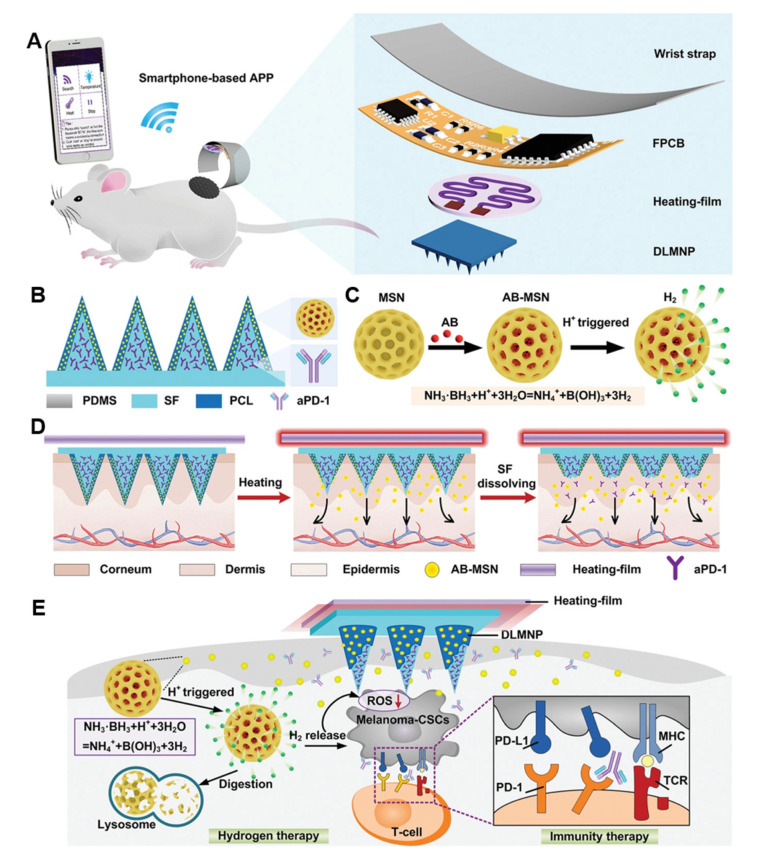
The schematic diagram of SMND preparation and the intelligent drug delivery scheme. (**A**) The SMND patch is mainly composed of a microneedle patch layer, a heating layer, and a flexible circuit layer (FPCB), which is controlled by a smartphone application (APP). (**B**) The structure and composition of the microneedle patch layer. The SF loaded with aPD-1 is inside the microneedle, and the PCA-coated AB-MSN is a thermally responsive outer coating. (**C**) The acid-triggered decomposition and release H2 diagram of AB-MSN. (**D**) The drug release diagram of SMND under APP control. (**E**) The mechanism of SMND synergistic immune/hydrogen therapy for CSCs. Reprinted with permission from [167]. Copyright 2022, John Wiley and Sons.

Cancer stem cells (CSCs) are the main cause of tumor recurrence, progression, and treatment failure [159,161,162,163,164]. Although aPD1 antibody can be used to treat CSCs, its therapeutic effect is greatly reduced due to the immune escape evolution of CSCs [165]. In one study, a wearable silk microneedle device (SMND) was developed combining synergistic immunization and hydrogen therapy. The device is made of a double-layer microneedle patch (DLMNP) and drug-loaded APD1 silk fibroin (SF) as an internal matrix for immunotherapy [166]. Ammoniaborane-loaded mesoporous silica nanoparticle (AB-MSN) coated polycaprolactone releases hydrogen in the layer upon thermal stimulation. The highlight of SMND is that the release of responsive drugs can be controlled by smartphones to achieve sustained anti-CSC therapy (Figure 12). The experimental results of the melanoma model using B16F10-CSCs tumor-bearing mice showed that the synergistic treatment strategy had minimal systemic toxicity and could achieve satisfactory antitumor and anti-CSCs effects. In summary, the intelligent and collaborative design of SMND has elevated the diversity and controllability of microneedle-based immunotherapy treatment methods to a new level.

Table 3 lists several of the immune microneedle anticancer vaccines with practical value and market prospects, outlining their design ideas, response methods, and applicable diseases. We hope to provide effective ideas for the design of anticancer microneedle-based immunotherapy.

## 5. The Limitations of the Microneedle-Based Drug Delivery System

Although the microneedle-based drug delivery systems have many advantages and market application prospects, some physical and chemical limitations still need to be addressed in future. The molecular weight of the drug planned for use in the microneedle-based drug delivery system should be less than 500 Da [174], and the lipophilic LogP should be 1–3 in order to diffuse smoothly in the skin [175]. Therefore, the application of peptides and macromolecular drugs in microneedle drug delivery systems may be limited. Although bionic needle tips and precision 3D printing can improve the skin adhesion of microneedle patches, they are also accompanied by high equipment costs and optimal needle tip configurations that have not yet been clinically approved.

Dissolvable microneedles account for a large proportion of microneedle drug delivery. Because most of the needle tip materials are biodegradable polymers, it is necessary to uniformly disperse the drug in the microneedle or encapsulate the drug in the hollow needle tip, so the one-time administration dose is limited. Moreover, due to the characteristics of biocompatibility, dissolvable microneedles are not easy to preserve, and drugs can easily lose their activity, which greatly increases the preservation cost and limits production.

There are also issues such as the drug loading of the microneedle transdermal drug delivery devices, the stability of biological agents [176], and the unpredictable drug release rate in the skin [156], which require further investigation by researchers and pharmaceutical companies. Moreover, the time from production to final treatment is several months or so. Therefore, the microneedle needs to have stable mechanical strength, and the payload must remain safe and active. Pretreatment sterilization methods such as high temperature and ultraviolet irradiation can reduce the active ingredients of the immunotherapeutic agents. A micromechanical tester can be used to test the performance of microneedles with controllable mechanical force and compression time. Vaccines can be administered to suitable animals and then further characterized for immune responses. Overall, proper storage methods can greatly preserve the performance of microneedle-based immunotherapy.

## 6. Future Perspectives and Clinical Translation

Responsive microneedle-based immunotherapy combines multiple responses and multiple therapies, providing new possibilities for the precise treatment of diseases and the reduction of physical side effects from the perspective of materials and technology. This type of therapy overcomes the shortcomings of traditional drug carriers, can accurately control the drug release site, and can increase the therapeutic effect on diseases. Thus far, 143 clinical applications of microneedles have been reported: 47 on skin diseases, 15 on vaccines, 9 on diabetes, and 9 on eye diseases. There are many scientific studies being conducted on responsive microneedles, but the transfer to clinical application has been very limited. Researchers continue to explore methods of internal and external stimulation according to the needs of enhancing immunotherapy. Because single-response microneedles are sometimes insensitive to a single factor, researchers are currently focusing on the design of dual-response and multiresponse nanodrug carriers. Whether they will provide better vaccine delivery or immune system activation requires more research and exploration to determine. As further research overcomes hurdles, microneedle-based immunotherapy delivery could play an important role in the way vaccinations are delivered in the future.

## Figures and Tables

**Table 1:** Representative responsive microneedle triggered by internal stimuli.

Design of Microneedle-Based Immunotherapy	Stimulus	Payload	Application	Ref.
H_2_O_2_-labile and positively charged amphiphilic diblock copolymer	Glucose/H_2_O_2_/pH	Insulin	Diabetes	[102]
Dimethylmaleic anhydride-modified polylysine	pH	p53 DNA	Cancer therapy	[66]
Hyaluronic acid (HA) = integrated dextran nanoparticles	pH	aPD1	Cancer immunotherapy	[56]
Gelatin methacrylate (GelMa)/4-(2-acrylamidoethylcarbamoyl)-3-fluorophenylboronic acid (AFPBA)	Glucose	Gluconic insulin	Diabetic wound	[55]
1-methyl-DL-tryptophan-conjugated HA	Hyaluronidase (HAase)	Anti-PD1 antibody (aPD1)	Cancer immunotherapy	[103]
Methacrylated hyaluronic acid (MeHA)	Low glucose	Glucagon	Hypoglycemia	[104]
Biomass chitosan microneedle array (CSMNA) patch	Temperature	Vascular endothelial growth factor	Wound healing	[47]
Polyvinyl alcohol as the tip and hyaluronic acid/diatomaceous earth as the base	ROS	Clindamycin	Acne vulgaris	[81]
Gelatin methacrylate (GelMa)/4-(2-acrylamidoethylcarbamoyl)-3-fluorophenylboronic acid (AFPBA)	Glucose	Gluconic insulin	Diabetic wound	[55]

**Table 2:** Representative responsive microneedle-based immunotherapy triggered by external stimulus.

Design of Microneedle-Based Immunotherapy	Stimulus	Payload	Application	Ref.
Dissolvable hyaluronic acid (HA) tips and biocompatible polycaprolactone (PCL) bases	Mechanical stress	Antigens derived from canine influenza virus	Influenza vaccination	[117]
Photothermal black-phosphorus (BP) and phase-changing gelatin	Near-infrared	Interferon γ (IFN-γ) and Dexa-methasone	Lupus erythematosus treatment	[118]
Hyaluronic acid-based microneedles encapsulating tumor lysates, melanin, and adjuvants	Near-infrared	B16F10 whole tumor lysate	Cancer immunotherapy	[119]
Asymmetric microneedles loaded with *S. japonicum* egg tips on CA-CMC	Mechanical stress	*Schistosoma japonicum* egg	Type 1 diabetes mellitus	[120]
A double-sided adhesive impermeable gasket, MN arrays, and Ag/AgCl electrodes	Electric	Ovalbumin	COVID-19	[121]
Rolling structures and a microneedle electrode array	Electric	siRNA(PD-L1) alone or combined with aPD-1 or immunoadjuvant of CPG2395	c\Cancer immunotherapy	[2]
Monomethoxy-poly (ethylene glycol)-polycaprolactone-loaded HA	Near-infrared	5-fluorouracil (5-Fu)	Cancer therapy	[105]
Homologous zeolitic imidazolate framework-8 (ZIF-8)	Near-infrared	Glucose oxidase/catalase (CAT)	Cancer therapy	[122]
Poly(vinylpyrrolidone) (PVP) as a substrate	Near-infrared	CuO2 nanoparticles	Cancer therapy	[123]
PEGylated gold nanorod (GNR-PEG) coated poly (L-lactide)	Near-infrared	Docetaxel	Cancer therapy	[114]
Polyvinyl acetate (PVA) as base/gelatin methacryloyl (GelMA) as tip	Near-infrared	Black phosphorus/quantum dots	Wound healing	[124]
Polycaprolactone	Thermal stimulus	Metformin	Diabetes	[125]
2-hydroxyethyl methacrylate and ethylene glycol Di methacrylate (EGDMA)	UV radiation	Ibuprofen	Pain relief	[126]
A magnetically responsive microneedle robot manufactured with 3D printing and encapsulation in commercial enteric capsules.	Magnetic fields	Insulin	Diabetes	[127]

**Table 3:** Representative responsive microneedle vaccine for cancer immunotherapy.

Design of Microneedle-Based Immunotherapy	Stimulus	Payload	Application	Ref.
Ammonia borane-loaded mesoporous silica nanoparticle (AB-MSN)-encapsulated polycaprolactone	Thermal	aPD-1-loaded silk fibroin (SF)	Melanoma immunotherapy	[167]
Hyaluronic acid	Near-infrared	Tumor lysate containing melanin	Melanoma immunotherapy	[168]
Polyvinyl acetate (PVA)	Skin environment soluble	RALA/pDNA nanoparticles	TRAMP-C1 tumors	[169]
Cryomicroneedles (cryoMNs)	Skin temperature soluble	Dendritic cell (DC)/aPD1	Melanoma immunotherapy	[170]
Positively charged ultra-pH-responsive oligo sulfamethazine conjugated poly (β-amino ester urethane) (OSM-(PEG-PAEU)) and negatively charged immunostimulatory adjuvant poly(I:C)	pH	Immunostimulatory adjuvant poly(I:C)/DNA polyplex	Melanoma immunotherapy	[42]
Mannosylated N,N,N-trimethyl chitosan (mTMC) as the gene vector and paclitaxel as the adjuvant	Skin environment soluble	Melanoma antigen tyrosinase-related protein-2 (Trp-2)	Melanoma immunotherapy	[171]
Hyaluronic acid	NIR irradiation	Immunogenic cell death-inducer (IR780) and autophagy inhibitor (chloroquine, CQ) coencapsulated micelles (C/I-Mil)	Photoimmunotherapy	[172]
Biocompatible hyaluronic acid integrated with the pH-sensitive dextran nanoparticles	pH/UV light	Checkpoint inhibitor anti-CTLA4 antibody (aCTLA4)	4T1 models photodynamic/immunotherapy	[173]

## Data Availability

Not applicable.
